# Epidemiological Characteristics of Notifiable Respiratory Infectious Diseases in Mainland China from 2010 to 2018

**DOI:** 10.3390/ijerph20053946

**Published:** 2023-02-23

**Authors:** Lele Deng, Yajun Han, Jinlong Wang, Haican Liu, Guilian Li, Dayan Wang, Guangxue He

**Affiliations:** 1 National Institute for Viral Disease Control and Prevention, Chinese Center for Disease Control and Prevention, Beijing 102206, China; 2 State Key Laboratory for Infectious Disease Prevention and Control, Collaborative Innovation Center for Diagnosis and Treatment of Infectious Diseases, National Institute for Communicable Disease Control and Prevention, Chinese Center for Disease Control and Prevention, Beijing 102206, China

**Keywords:** respiratory infectious diseases, epidemiological characteristics, incidence, mortality, spatiotemporal analysis, temporal distribution, seasonal distribution, China

## Abstract

Respiratory infectious diseases (RIDs) pose threats to people’s health, some of which are serious public health problems. The aim of our study was to explore epidemic situations regarding notifiable RIDs and the epidemiological characteristics of the six most common RIDs in mainland China. We first collected the surveillance data of all 12 statutory notifiable RIDs for 31 provinces in mainland China that reported between 2010 and 2018, and then the six most prevalent RIDs were selected to analyze their temporal, seasonal, spatiotemporal and population distribution characteristics. From 2010 to 2018, there were 13,985,040 notifiable cases and 25,548 deaths from RIDs in mainland China. The incidence rate of RIDs increased from 109.85/100,000 in 2010 to 140.85/100,000 in 2018. The mortality from RIDs ranged from 0.18/100,000 to 0.24/100,000. The most common RIDs in class B were pulmonary tuberculosis (PTB), pertussis, and measles, while those in class C were seasonal influenza, mumps and rubella. From 2010 to 2018, the incidence rate of PTB and rubella decreased; however, pertussis and seasonal influenza increased, with irregular changes in measles and mumps. The mortality from PTB increased from 2015 to 2018, and the mortality from seasonal influenza changed irregularly. PTB was mainly prevalent among people over 15 years old, while the other five common RIDs mostly occurred among people younger than 15 years old. The incidence of the six common RIDs mostly occurred in winter and spring, and they were spatiotemporally clustered in different areas and periods. In conclusion, PTB, seasonal influenza and mumps remain as public health problems in China, suggesting that continuous government input, more precise interventions, and a high-tech digital/intelligent surveillance and warning system are required to rapidly identify emerging events and timely response.

## 1. Introduction

Respiratory infectious diseases (RIDs) pose threats to people’s health and lives, some of which are serious public health problems. RIDs are known to spread mainly through droplets and short-distance contact [1], and people are generally susceptible to the pathogens of RIDs. It is estimated that there are 1 billion seasonal influenza cases worldwide every year, of which 3–5 million are severe cases and 290,000–650,000 lead to influenza-related respiratory deaths [2]. Pulmonary tuberculosis (PTB) is listed as one of the top 10 causes of death worldwide by a single infection [3]. Furthermore, the emerging RIDs that have occurred recently, related to coronavirus disease 2019 (COVID-19), remind us that RIDs are always significant concerns and need more effective surveillance. Therefore, it is meaningful to characterize the temporal, seasonal, spatiotemporal and population distribution characteristics of these RIDs in order to make precise interventions and more effective control measures.

Many studies have explored the epidemiological characteristics of infectious diseases in China [4,5,6,7]. For example, Jiang et al. analyzed the epidemiological characteristics and trends of notifiable infectious diseases in China from 1986 to 2016 [4]. Dong et al. reported the trends and variations between age groups, sex, seasons and provinces of the infectious diseases persisting in children and adolescents in China from 2008 to 2017 [5]. Moreover, several studies analyzing the spatial–temporal distributions of single RIDs, such as tuberculosis [6] and influenza [7], have been reported. However, most previous studies focused on all or single notifiable infectious diseases within various time intervals, and an updated and systematic analysis of the epidemiological characteristics of RIDs in China is lacking. In this study, we explored the overall epidemic situation of all 12 statutory notifiable RIDs and the epidemiological characteristics (temporal, seasonal, spatiotemporal and population distributions) of the six most common RIDs in mainland China.

## 2. Materials and Methods

### 2.1. Data Sources

Data between 2010 and 2018 were collected from the monthly and regional reports from the China Public Health Science Statistics Center (http://www.phsciencedata.cn, accessed on 10 March 2022) of the Chinese Center for Disease Control and Prevention [8]. All notifiable infectious disease cases were reported on the China Information System for Disease Control and Prevention (CISDCP) of the Chinese Center for Disease Control and Prevention, and surveillance data were published on the above websites. The standard population data used in the Joinpoint regression analysis was from the Chinese Population Census in 2010 [9]. The standard base map of China [GS(2019)1822] was from the Standard Map Service (http://bzdt.ch.mnr.gov.cn/, accessed on 20 February 2023) of the Ministry of Natural Resources of the People’s Republic of China.

### 2.2. Classification of RIDs and Studied Regions

Notifiable infectious diseases were classified into three types in China, namely class A, B, and C. A total of 12 notifiable RIDs were included in this study, eight RIDs from class B (PTB, pertussis, measles, epidemic cerebrospinal meningitis, scarlet fever, highly pathogenic avian influenza (HPAI), H7N9 influenza and diphtheria) and four RIDs from class C (seasonal influenza, influenza A (H1N1), mumps and rubella). Influenza A (H1N1) was classified into class C and combined with seasonal influenza in 2013 [10].

Thirty-one provinces in mainland China are divided into seven areas according to geographic location, population, environment, and other factors: northern China (Beijing, Tianjin, Hebei, Shanxi and Inner Mongolia), northeastern China (Heilongjiang, Jilin and Liaoning), eastern China (Shandong, Jiangsu, Shanghai, Zhejiang, Anhui, Jiangxi and Fujian), central China (Henan, Hubei and Hunan), southern China (Guangdong, Guangxi and Hainan), southwestern China (Sichuan, Yunnan, Guizhou, Chongqing and Tibet), and northwestern China (Xinjiang, Ningxia, Qinghai, Shaanxi and Gansu).

### 2.3. Data Analysis

We first analyzed the overall epidemic situation of 12 RIDs from 2010 to 2018, then focused on the top three RIDs from class B (PTB, pertussis and measles) and the top three RIDs from class C (seasonal influenza, mumps and rubella) with the highest incidence rates from 2010 to 2018 in mainland China, to analyze age-specific rates, temporal trends, and seasonal and spatiotemporal clustering characteristics. The deaths caused by most RIDs are sporadic, so we only analyzed the deaths related to PTB and seasonal influenza, which caused higher mortality.

Descriptive analysis was used to present the population distribution characteristics of RIDs, and the figure drawing was conducted with OriginPro (version 2021, OriginLab Corporation, Northampton, MA, USA).

### 2.4. Joinpoint Regression Model

The temporal trends of incidence rate and mortality were characterized by annual percentage changes (APCs) and were estimated with Joinpoint regression software (version 4.9.0.0, National Cancer Institute, Rockville, MD, USA). The grid search method (GSM) was the default modeling method, and the Monte Carlo permutation test was the default optimization method of the model. The Bayesian information criterion (BIC) was used to correct the statistically significant level. The most appropriate joinpoints of the interval piecewise function were finally selected by the above methods [11]. The age-standardized incidence rate and mortality were used in this study. The rate trends were considered to increase if APC > 0 (*p* < 0.05) and to decrease if APC < 0 (*p* < 0.05), otherwise rates were considered stable [6]. The figures for temporal trends were conducted by Joinpoint regression software (version 4.9.0.0, National Cancer Institute, Rockville, MD, USA).

### 2.5. Seasonal Decomposition

Seasonal decomposition of time series by the LOESS method for extracting components was used to assess the seasonal pattern of the data. The decomposition breaks the time series of RIDs incidences into three components: Yt = Tt + St + Rt [1,12]. Here, Yt represents the monthly rate of the diseases. Tt represents a trend component, which reflects the long-term trend. St represents the seasonal component, reflecting seasonality in a fixed period. Rt represents the remainder component, which describes the residuals of the time series [1]. Seasonal analysis and figure drawing was conducted by SPSS (version 20.0, IBM Inc., Armonk, NY, USA).

### 2.6. Spatiotemporal Clustering Analysis

SaTScan (version 10.1, Kulldorff and Information Management Services, Inc., Boston, MA, USA) was used to identify the space–time clusters of RIDs in different areas and during different periods based on the Poisson probability model [13,14]. The scanning window is a time interval for a cylinder in the space–time scan, with a spatial window for 30% of the study period and a temporal window for 50% [14]. SaTScan gradually scans time and space, comparing observed and predicted events (assuming random distribution) in each location window to identify likely clusters. The cluster with the highest log-likelihood ratio (LLR) is considered the most likely cluster, and the others are secondary clusters in order [13]. Mapping of the spatiotemporal features was conducted by ArcGIS software (version 10.7, ESRI, Redlands, CA, USA).

## 3. Results

### 3.1. Overall Epidemic Situation of RIDs

From 2010 to 2018, there were 13,985,040 notifiable cases of RIDs in mainland China. The incidence rate of RIDs increased from 109.85/100,000 in 2010 to 140.85/100,000 in 2018. PTB was the most prevalent RIDs and accounted for 42.08–67.62% of notifiable cases between 2010 and 2018. During the period 2010 to 2018, PTB had the highest average annual incidence rate (65.79/100,000), followed by mumps (21.40/100,000), seasonal influenza (19.22/100,000), scarlet fever (4.09/100,000), measles (1.73/100,000), rubella (1.61/100,000), pertussis (0.46/100,000) and epidemic cerebrospinal meningitis (0.01/100,000). The incidence of HPAI and H7N9 were extremely low: there were only 14 notifiable cases of HPAI during 2010–2015 and 1385 notifiable cases of H7N9 seasonal influenza, which mainly occurred from 2013 to 2017. No diphtheria cases were reported (Table 1).

During the 9 years, 25,548 deaths for 12 RIDs were reported, and the range of mortality was from 0.18/100,000 to 0.24/100,000. PTB accounted for 89.70–94.96% of RIDs death cases, ranking as first, followed by seasonal influenza (0.26~4.61%). There were 919 reported deaths from the other ten RIDs during the 9 years, including H7N9 influenza (560 deaths), epidemic cerebrospinal meningitis (167 deaths), measles (153 deaths), pertussis (13 deaths), HPAI (10 deaths), mumps (seven deaths), scarlet fever (six deaths), and rubella (three deaths).

### 3.2. Age-Specific Incidence Rates and Mortalities for the Six Selected RIDs

**Figure 1:**
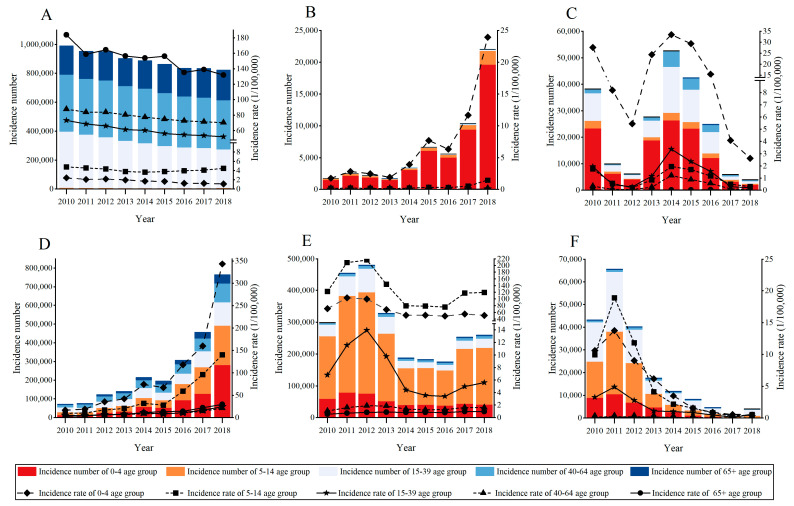
Age-specific incidence cases and rates of the six selected RIDs from 2010 to 2018. Note: (**A**) pulmonary tuberculosis; (**B**) pertussis; (**C**) measles; (**D**) seasonal influenza; (**E**) mumps; (**F**) rubella.

The age-specific incidence rates of the six RIDs are shown in Figure 1. For PTB, the highest age-specific incidence rate was in the group of people aged 65 years old and over, and varied from 183.78/100,000 in 2010 to 131.81/100,000 in 2018 (Figure 1A). However, our collected data showed that notifiable cases of PTB were the most in the group aged 40–64 and accounted for 39.68% to 42.39% during 2010 to 2018. Pertussis was mostly observed to occur in children, and the 0–4 age group carried the highest incidence rate (1.75/100,000 in 2010 to 23.92/100,000 in 2018) and the majority of incidence cases (accounting for 84.47% to 91.90% during 2010 to 2018) (Figure 1B). For measles, the 0–4 age group showed the highest incidence cases and rate, followed by the 15–39 age group (Figure 1C). The most incidence cases and the highest rate of seasonal influenza were both in the 0–4 age group, followed by the 5–14 age group (Figure 1D). In contrast to seasonal influenza, the most incidence cases and the highest rate of mumps were both in the 5–14 age group, followed by the 0–4 age group (Figure 1E). The incidence cases of rubella were mostly found in the 15–39 age group, but the highest incidence rate was found in people less than 15 years old (Figure 1F).

**Figure 2:**
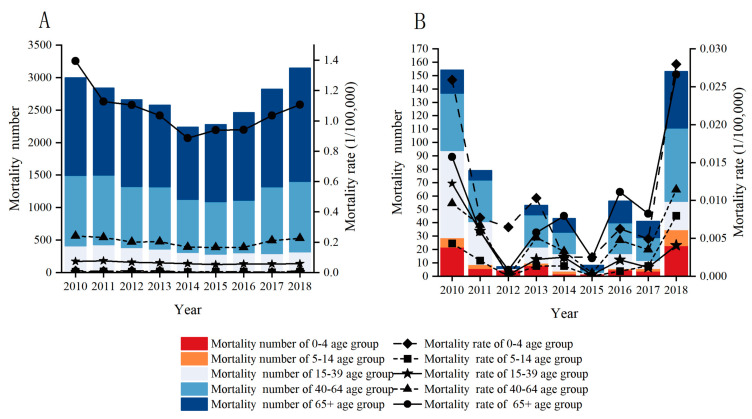
Age-specific mortality number and rates of pulmonary tuberculosis and seasonal influenza from 2010 to 2018. Note: (**A**) pulmonary tuberculosis; (**B**) seasonal influenza.

Due to the relatively higher burden posed by PTB and seasonal influenza among the six selected RIDs, we only analyzed the age-specific mortalities of these two RIDs (Figure 2). We found that reported deaths and mortality for the PTB both increased with age, and the highest were both for people aged 65 and over (Figure 2A). For seasonal influenza, the highest reported deaths were in the 40–64 age group, while mortality was more frequent in the 0–4 age group and 65+ years groups (Figure 2B).

### 3.3. Temporal Trends of Incidence Rates and Mortalities of the Six Selected RIDs

**Figure 3:**
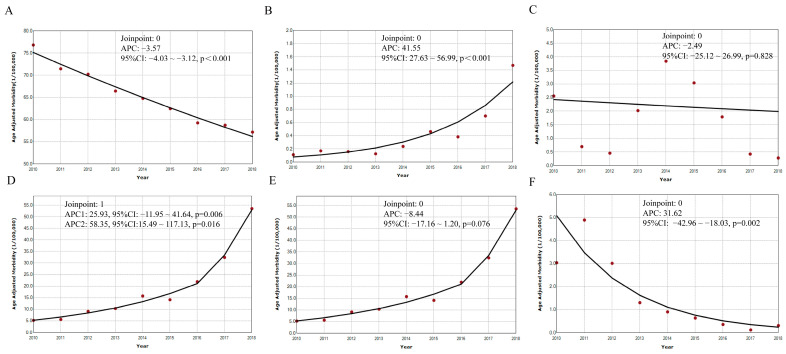
Temporal trends for incidence rates of the six selected RIDs from 2010 to 2018. Note: (**A**) pulmonary tuberculosis; (**B**) pertussis; (**C**) measles; (**D**) seasonal influenza; (**E**) mumps; (**F**) rubella. APC annual percentage change. CI confidence interval.

The temporal trends of the age standardized incidence rates of the six selected RIDs are shown in Figure 3. We observed that the six RIDs showed different temporal trends from 2010 to 2018. The age standardized incidence rate of PTB declined yearly from 76.81/100,000 in 2010 to 57.16/100,000 in 2018, with −3.57% APC (95% CI: −4.03~−3.12, *p* < 0.001) (Figure 3A), while the age standardized incidence rate of pertussis increased from 0.11/100,000 in 2010 to 1.47/100,000 in 2018, with APC at 41.55% (95% CI: 27.63~56.99, *p* < 0.001) (Figure 3B); for measles and mumps, both age standardized incidence rates remained stable over the nine years with APCs of −2.49% (95% CI: −25.12~26.99, *p* = 0.828) (Figure 3C) and −8.44% (95% CI: −17.16~1.20, *p* = 0.076) (Figure 3E), respectively; and the age standardized incidence rate of rubella declined, with APC of −31.62% (95% CI: −42.96~−18.03, *p* = 0.002) (Figure 3F). For seasonal influenza, one joinpoint was identified in 2016, and the age standardized incidence rate increased with APC of 25.93% (95% CI: 11.95~41.64, *p* = 0.006) from 2010 to 2016, and increased with APC of 58.35% (95 %CI: 15.49~117.13, *p* = 0.016) from 2016 to 2018 (Figure 3D).

**Figure 4:**
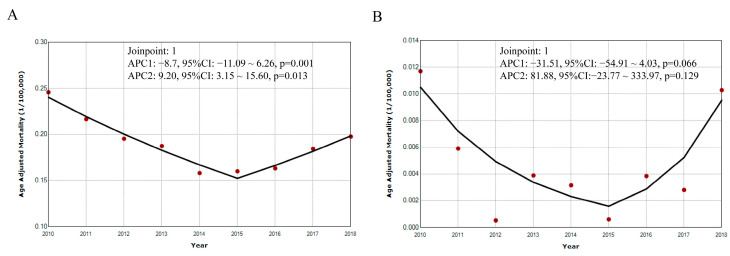
Temporal trends of mortalities for pulmonary tuberculosis and seasonal influenza from 2010 to 2018. Note: (**A**) pulmonary tuberculosis; (**B**) seasonal influenza. APC annual percentage change. CI confidence interval.

We also analyzed the temporal trends of the age standardized mortalities of PTB and seasonal influenza (Figure 4). For PTB, the age standardized mortality declined from 0.25/100,000 in 2010 to 0.20/100,000 in 2018, and one joinpoint was found in 2015; from 2010 to 2015 the APC declined by −8.71% (95% CI: −11.09~6.26, *p* = 0.001) and increased by 9.20% (95% CI: 3.15~15.60, *p* = 0.013) from 2015 to 2018 (Figure 4A). For seasonal influenza, the age standardized mortality remained stable across the decades, although one joinpoint was identified in 2015, with APCs of −31.51% (95% CI: −54.91~4.03, *p* = 0.066) from 2010 to 2015 and 81.88% (95% CI: −23.77~333.97, *p* = 0.129) from 2015 to 2018 (Figure 4B).

### 3.4. Seasonal Distributions of Incidence Rates of the Six Selected RIDs

**Figure 5:**
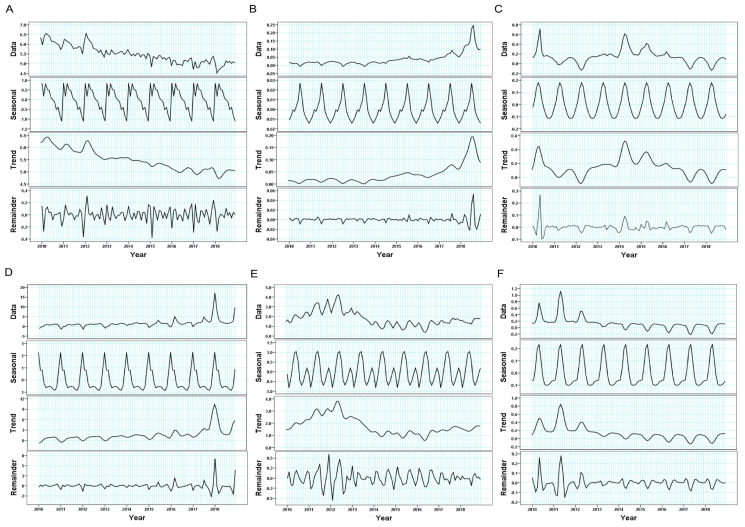
Seasonal decomposition of monthly incidence rates of the six selected RIDs. Note: (**A**) pulmonary tuberculosis; (**B**) pertussis; (**C**) measles; (**D**) seasonal influenza; (**E**) mumps; (**F**) rubella. Data represents the time series adjusted by seasonal components. Seasonal represents seasonality decomposed from time series. Trend represents the long-term progression and repeated but non-periodic fluctuations of series. Remainder represents residuals of the time series.

The results from seasonal decomposition analysis show that almost all of the six RIDs occurred in winter or spring, and each has its own characteristics (Figure 5). The incidence rate for PTB presented two peaks in Nov. and Mar., respectively (Figure 5A). The high-incidence seasons for seasonal influenza were in Dec., Jan., Feb. and Mar., with a peak in Jan. (Figure 5D). We then found an interesting phenomenon—that the peak-incidence seasons of the other four RIDs appeared in sequence after the influenza seasons. For example, measles showed peak incidence in Apr. (higher incidence in Mar., Apr. and May, Figure 5C), rubella in May. (higher incidence in Apr., May and Jun., Figure 5F), mumps in Jun. (higher incidence in May, Jun. and Jul., Figure 5E), and pertussis in Jul. (higher incidence in Jun., Jul. and Aug., Figure 5B).

### 3.5. Spatial Distribution of Incidence Rates for the Six Selected RIDs

In most provinces of mainland China, the majority of RIDs incidence trends were consistent with the national trend, with only a few provinces showing opposite trends in the incidence of a few RIDs (Table 2). The incidences of the six selected RIDs showed different spatial distributions in the 31 provinces. Compared to 2010, the incidence rates of PTB in 2018 decreased in 27 provinces, with the greatest decrease in Gansu (57.30%) in northwestern China, and increased in four provinces, including Tibet, Qinghai, Yunnan and Xinjiang, with percentage change of 40.84%, 60.65%, 7.21% and 85.42%, respectively. The incidence rate of pertussis showed a rebound in most provinces of China. The incidence rate of measles decreased and remained low in most provinces. By comparing to 2010, three provinces, Yunnan, Hainan, and Fujian, showed increases in the incidence rate of measles in 2018, while the whole country showed a decreasing trend. Seasonal influenza has been prevalent throughout the country, especially in southern, central, eastern and northern China in recent years. The incidence rate of seasonal influenza increased in 29 provinces, with the greatest increase in Beijing, Hubei, Guangdong and Zhejiang. The incidence rate of mumps decreased in 16 out of 31 provinces, and the largest percentage change was 79.34% in Ningxia; it increased in 15 provinces, with the greatest change found in Tibet (107.38%), followed by Hunan (46.36%) and Qinghai (46.58%). The incidence rate of rubella remained at a low level in the whole country. Compared to 2010, the incidence rate of rubella decreased in 29 out of 31 provinces, but increased in Hainan (324.94%) and Guangxi (9.95%).

### 3.6. Spatiotemporal Clustering Analysis of Incidence Rates and Mortalities for the Six Selected RIDs

**Figure 6:**
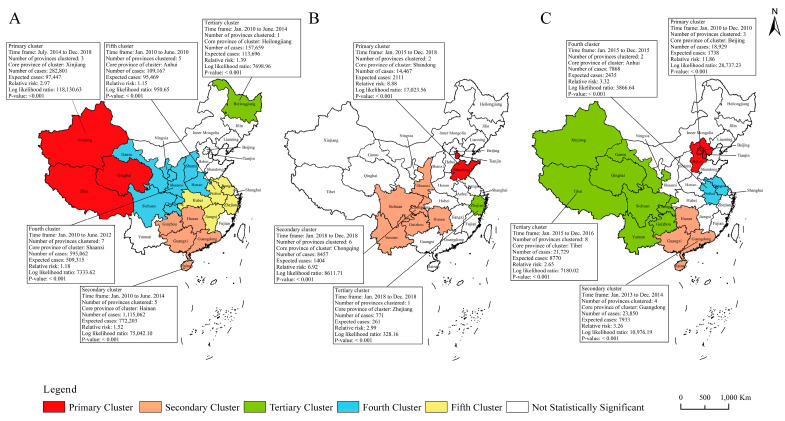
Spatiotemporal clustering of incidence rates for three RIDs of class B in 31 provinces of mainland China. Note: (**A**) pulmonary tuberculosis; (**B**) pertussis; (**C**) measles.

**Figure 7:**
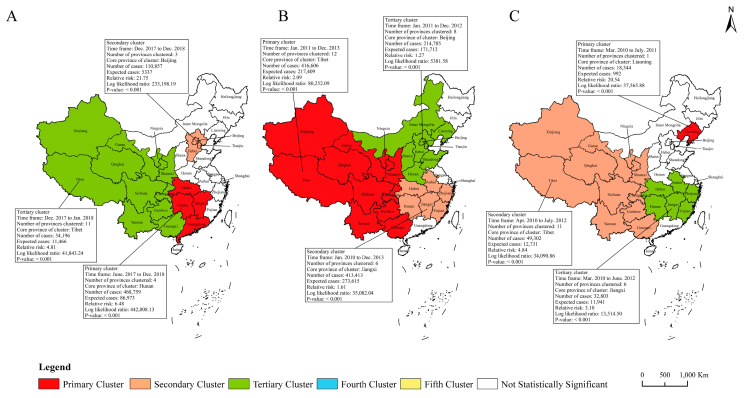
Spatiotemporal clustering of incidence rates for three class C RIDs in 31 provinces of mainland China. Note: (**A**) seasonal influenza; (**B**) mumps; (**C**) rubella.

The spatiotemporal clustering analysis showed that the incidences of RIDs was spatiotemporally clustered (Figure 6 and Figure 7). The incidence of PTB showed five clusters, measles four clusters, and each of pertussis, seasonal influenza, mumps and rubella three clusters, respectively. For PTB, the five clusters of PTB incidence covered 21 provinces, and the most likely cluster was mainly distributed in northwestern China and covered three provinces during Jul. 2014 to Dec. 2018, with a relative risk (RR) of 2.97 (*p* < 0.001). The other four clusters of PTB were before 2014 (Figure 6A). The three clusters of pertussis incidence covered nine provinces, mainly occurring in 2018, and one primary cluster distributed in Shandong and Tianjin during Jan. 2015 to Dec. 2018, with RR of 8.88 (*p* < 0.001) (Figure 6B). For measles, the four clusters covered 17 provinces in different periods, and the primary cluster covered three provinces in 2010, with RR of 11.86 (*p* < 0.001) (Figure 6C). The three clusters of seasonal influenza covered 18 provinces during 2017–2018, and the primary cluster was mainly distributed in the eastern and central China, with RR of 6.48 (*p* < 0.001) (Figure 7A). The three clusters for mumps incidence covered 26 provinces before 2013, and the primary cluster covered 12 provinces, mainly located in western China, with RR of 2.09 (*p* < 0.001) (Figure 7B). The three clusters of rubella incidence covered 18 provinces before 2012, and the primary cluster was located in Liaoning during Mar. 2010 to Jul. 2011, with RR of 20.54 (*p* < 0.001) (Figure 7C).

**Figure 8:**
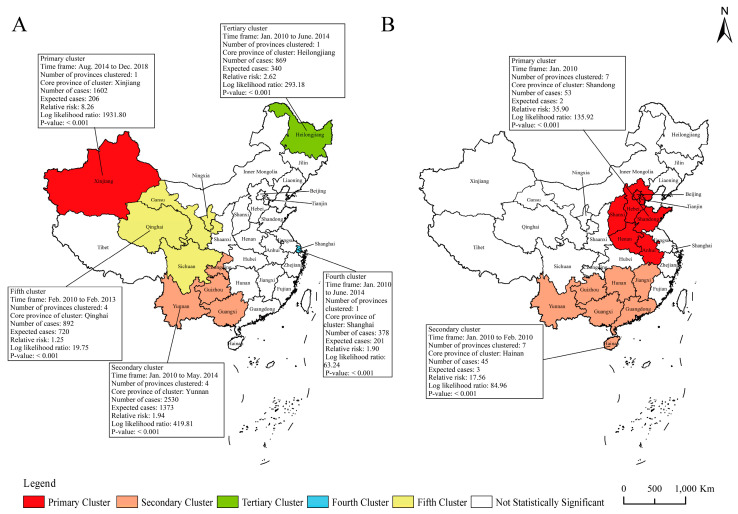
Spatiotemporal clustering of mortalities for pulmonary tuberculosis and seasonal influenza in 31 provinces of mainland China. Note: (**A**) pulmonary tuberculosis; (**B**) seasonal influenza.

Similarly, the mortality for PTB and seasonal influenza all showed spatiotemporal clustering (Figure 8). The five clusters for PTB mortality covered 10 provinces, and the primary cluster was located in Xinjiang during Aug. 2014 to Dec. 2018, with RR of 8.26 (*p* < 0.001) (Figure 8A). The other four clusters of PTB mortality mainly occurred before 2014. For seasonal influenza, the two clusters covered 14 provinces during Jan. 2010 to Feb. 2010, and the primary cluster covered seven provinces, with Shandong as the center.

## 4. Discussion

Infectious diseases are serious public health problems affecting a large portion of the population all over the world. The Chinese government has attached great importance to and is strongly committing to the control of infectious diseases. National surveillance for the selected infectious diseases in China started from a paper-based reporting system in the 1950s, moving to an electronic file-based system in 1985 and to a web-based reporting system (CISDCP) since 2003 [15]. By analyzing the surveillance data, we first studied the overall epidemiological characteristics of the all notifiable RIDs in mainland China and analyzed up-dated epidemiology characteristics of six common RIDs.

Our results showed that some kinds of RIDs were already at low incidence levels (pertussis, measles and rubella) or had even basically been eliminated (diphtheria and epidemic cerebrospinal meningitis). However, given increased economic globalization, urbanization, mobility and the emergence of drug-resistance, the incidences of some RIDs (PTB, seasonal influenza and mumps) showed dumps, or were still at high levels in some areas of China. In the present study, we particularly analyzed the epidemiological characteristics of the six most common RIDs in mainland China.

The present study showed that the morbidity for PTB in mainland China has been decreasing yearly, which is similar to the global trend [3] and may be due to the broad adoption of the directly observed treatment short-course strategy (DOTS), the Stop TB Strategy, the End TB Strategy and mass public health interventions in China [16]. However, China is still one of the 30 countries with a high burden of PTB [3]. We found that the PTB burden in the western areas (Qinghai, Tibet and Xinjiang) of China was much higher than in the other areas of China during 2014–2018. It is known that tuberculosis is a disease associated with poverty [3]. The poor health resources in the western provinces of China may lead to delayed detection and diagnosis, as well as a poor health service and patient management [17]. Moreover, the lack of self-protection awareness of the residents in these regions may be associated with the spread of PTB [18]. Furthermore, the improving coverage rate of CISDCP and the increasing applications of new diagnosis techniques (such as molecular tests) in the health care facilities in the western areas may be associated with the increased incidence rates of PTB in these provinces [5]. By age-specific analysis, we found that adults were the main population attacked by PTB, especially people aged 65 years old and over, which was in line with a previous study [19]. Due to the rapidly ageing population and longer life expectancy in China [20], the key population for PTB control in China may shift from younger to elderly populations in the future [19]. We also found that the incidence rate of PTB peaked in Nov. and Mar., suggesting that climatic factors and human activities in different seasons may affect incidence. The cold weather and more indoor activities in winter and spring increases the chance of infection, as well as crowded living and bad ventilation [21]. Therefore, it is important to increase financing and enhance the capacity of health facilities in high-risk areas for case detection, diagnosis and treatment. Moreover, we should pay more attention to high incidence seasons and populations and implement effective measures.

With the development of the health service, especially the great improvement in diagnostic ability and surveillance systems, the number of notifiable cases of seasonal influenza has increased year by year. In our study, the incidence of seasonal influenza mostly occurred in the population aged less than 15 years old. Previous studies showed that students and primary and secondary schools are high-risk groups for seasonal influenza, and specific places are prone to occur cluster epidemics [22,23,24]. As we know, winter and spring are the high incidence seasons for seasonal influenza. According to a systematic review, low temperatures and dry climatic conditions in winter and spring increase the chances of viral survival and transmission, subsequently leading to an increased incidence of influenza [25]. We observed that seasonal influenza is predominantly prevalent in the surrounding provinces of Hunan and Beijing during 2017–2018, with rapid growth of population density, accelerated population mobility, intensified urbanization [5], specific latitudes and geographic locations [26], and so on. Moreover, these provinces also have high medical accessibility, which facilitates the diagnosis of seasonal influenza. The mortality for seasonal influenza in mainland China went from 0.012/100,000 in 2010 to 0.011/100,000 in 2018. However, it is estimated that influenza-associated all-cause mortality was 14.33/100,000 in mainland China [27], which was much higher than in the present study. A previous study pointed out that the death burden from seasonal influenza had been underestimated, owing to the deaths from seasonal influenza and other respiratory diseases that are difficult to distinguish [2]. It is suggested that new measures should be adopted to strengthen seasonal influenza surveillance in key populations (students) and areas (provinces with high population density and mobility) in high-incidence seasons, and much more effective interventions should be taken.

Pertussis, measles, rubella and mumps are all vaccine-preventable diseases and mainly attack people under 15 years old. The peak-incidence months for measles, rubella, mumps and pertussis appear in sequence in spring and summer. However, the duration of peak period and temporal trends for the four RIDs were inconsistent. The incidence of pertussis in China has been decreasing due to the expanded program of immunization (EPI) [28], though its re-emergence has been noted in recent years. We found that the incidence of pertussis was spatiotemporally clustered and mainly occurred in 2018, and most provinces showed the re-emergence of pertussis in 2018, which is consistent with other studies [29,30]. The reasons for the re-emergence of pertussis remain unclear, and many factors affect the epidemiology of RIDs and need further exploration. For example, the variations in definitions and diagnostic criteria, the duration of immunity of the vaccine and the immunity level may influence the occurrence and spread of RIDs [31,32]. We should notice that epidemics of RIDs could not be explained by any single factor and should consider local conditions, which would lead to much more effective measures.

The incidence rate of measles and rubella remained at a low level in mainland China. The low incidence rates for measles and rubella reflected comprehensive preventative measures, including China’s Measles Elimination Action Plan [33], high sensitivity of surveillance and other measures to prevent and control these diseases [34]. Although the incidence rate for mumps spatiotemporally clustered during 2010–2013, it was still at a high level compared to measles and rubella. As we know, passive surveillance is the main tool to monitor infectious diseases in mainland China [35]. However, due to the limited laboratory detection technology in past decades, the diagnosis of some infectious diseases mainly depends on clinical diagnosis, which may lead to misdiagnosis. Notifiable cases of mumps are mostly confirmed by the clinical diagnostic criteria based on the “Diagnostic Criteria for Mumps (WS270-2007)” issued in 2007 [36]. With the development of detection technology, there are many laboratory detection methods which should be adopted for new disease diagnosis criteria for improving diagnosis accuracy and data reporting quality [37].

The present study demonstrated the achievement and challenges in the prevention and control of RIDs in mainland China, and called for continuous and increased efforts to prevent and control RIDs. We recommend more attention to projects or actions associated with strengthening the core capacity of public health, including enhancing government leadership and multiple sectors’ cooperation, expanding investment in prevention programs, enhancing and integrating digital/intelligent surveillance and warning systems, and strengthening pandemic preparedness [38]. Moreover, a variety of public health interventions implemented in China during the COVID-19 pandemic indicated potential benefits for the prevention of RIDs, such as social distancing, mask-wearing, prohibition of public gatherings and maintaining hand hygiene [35]. These behaviors should be advocated in further daily life. Due to data limitation, we only analyzed epidemiological characteristics from 2010 to 2018, and the quality of the report may be not consistent at different levels. Therefore, it is necessary to strengthen quality control of surveillance.

## 5. Conclusions

The incidence rate of RIDs increased from 2010 to 2018, and PTB, seasonal influenza and mumps were still at high levels. Some RIDs have been at low incidence levels (pertussis, measles and rubella) or almost eliminated (diphtheria and epidemic cerebrospinal meningitis). The study indicated that the incidence rate of PTB and rubella had decreased, but pertussis and seasonal influenza had increased, and there were irregular changes in measles and mumps. The mortality for PTB increased from 2015 to 2018, and the mortality for seasonal influenza changed irregularly. PTB was mainly prevalent among people over 15 years old, and the other five main RIDs mostly occurred in populations less than 15 years old. The incidence of the six RIDs mostly occurred in winter and spring. As for spatiotemporal clustering, the incidence and mortality of PTB were relatively higher in northwestern China during 2014–2018, while that of seasonal influenza clustered in most provinces during 2017–2018. The incidence rate of mumps clustered in most provinces, mainly occurring before 2013. In all, we still face big challenges and arduous tasks ahead for RIDs control and prevention in China and should take more precise and effective intervention, as well as establish new and effective high-tech digital/intelligent surveillance and warning platforms, to rapidly identify emergent events and instigate timely response.

## Figures and Tables

**Table 1:** The incidence of respiratory infectious diseases in mainland China from 2010–2018.

Year	Pulmonary Tuberculosis		Pertussis		Measles		Seasonal Influenza		Mumps		Rubella		Others		Total	
	Case (n)	Rate (1/100,000)	Case (n)	Rate (1/100,000)	Case (n)	Rate (1/100,000)	Case (n)	Rate (1/100,000)	Case (n)	Rate (1/100,000)	Case (n)	Rate (1/100,000)	Case (n)	Rate (1/100,000)	Case (n)	Rate (1/100,000)
2010	991,350	74.27	1764	0.13	38,159	2.86	71,625	5.37	298,932	22.40	43,117	3.23	21,202	1.59	1,466,149	109.85
2011	953,275	71.09	2517	0.19	9943	0.74	75,493	5.63	454,385	33.89	65,549	4.89	64,107	4.78	1,625,269	121.21
2012	951,508	70.62	2183	0.16	6183	0.46	123,212	9.15	479,518	35.59	40,156	2.98	46,655	3.46	1,649,415	122.42
2013	904,434	66.80	1712	0.13	27,646	2.04	140,714	10.39	327,759	24.21	17,580	1.30	34,430	2.54	1,454,275	107.40
2014	889,381	65.63	3408	0.25	52,628	3.88	215,533	15.91	187,500	13.84	11,793	0.87	54,745	4.04	1,414,988	104.41
2015	864,015	63.42	6658	0.49	42,361	3.11	195,723	14.37	182,833	13.42	8133	0.60	68,557	5.03	1,368,280	100.43
2016	836,236	61.00	5584	0.41	24,820	1.81	306,682	22.37	175,001	12.77	4535	0.33	59,647	4.35	1,412,505	103.04
2017	835,193	60.53	10,390	0.75	5941	0.43	456,718	33.10	252,740	18.32	1605	0.12	75,076	5.44	1,637,663	118.69
2018	823,342	59.27	22,057	1.59	3940	0.28	765,186	55.09	259,071	18.65	3930	0.28	78,970	5.69	1,956,496	140.85

Note: Others include epidemic cerebrospinal meningitis, scarlet fever, highly pathogenic avian, human infection with avian influenza A H7N9 virus. There was no diphtheria from 2010 to 2018.

**Table 2:** Incidence rates and percentage changes of respiratory infectious diseases in 31 provinces of mainland China.

Provinces and Regions	Pulmonary Tuberculosis			Pertussis			Measles			Seasonal Influenza			Mumps			Rubella		
	2010 (1/10,000)	2018 (1/10,000)	Percentage Change (%)	2010 (1/10,000)	2018 (1/10,000)	Percentage Change (%)	2010 (1/10,000)	2018 (1/10,000)	Percentage Change (%)	2010 (1/10,000)	2018 (1/10,000)	Percentage Change (%)	2010 (1/10,000)	2018 (1/10,000)	Percentage Change (%)	2010 (1/10,000)	2018 (1/10,000)	Percentage Change (%)
Beijing	45.70	30.43	−33.41	0.08	0.87	985.69	14.13	0.47	−96.64	6.37	381.84	5894.07	16.56	9.04	−45.41	6.21	0.27	−95.69
Tianjin	25.54	21.39	−16.26	0.99	5.27	430.87	16.22	0.42	−97.39	4.69	18.02	284.16	17.44	7.07	−59.49	6.71	0.12	−98.18
Hebei	59.08	39.31	−33.47	0.55	1.20	117.40	20.55	0.32	−98.44	19.63	74.21	278.10	16.17	18.60	15.00	4.87	0.04	−99.18
Shanxi	69.58	35.03	−49.66	0.18	0.49	182.35	0.70	0.08	−88.48	2.45	21.18	765.26	14.70	16.76	14.03	0.76	0.07	−90.35
Inner Mongolia	74.07	54.09	−26.98	0.02	0.19	819.52	2.83	0.27	−90.51	1.44	12.57	772.48	7.75	9.31	20.11	3.84	0.06	−98.46
Liaoning	60.10	55.81	−7.14	0.01	0.01	−20.91	0.96	0.05	−94.75	2.97	6.41	115.82	25.64	7.04	−72.54	19.64	0.04	−99.81
Jilin	83.79	47.43	−43.39	0.06	0.10	54.19	1.15	0.13	−89.08	2.76	9.79	255.19	16.29	5.84	−64.15	2.59	0.03	−98.72
Heilongjiang	95.84	65.23	−31.94	0.15	0.11	−26.87	16.46	0.25	−98.49	1.48	5.26	256.04	9.53	4.81	−49.51	1.16	0.02	−97.96
Shanghai	34.33	26.14	−23.87	0.00	0.47	-	1.34	0.24	−82.45	15.72	16.95	7.79	18.57	9.37	−49.56	1.45	0.09	−94.02
Jiangsu	53.93	31.02	−42.49	0.05	0.17	227.11	0.95	0.12	−86.89	3.56	14.67	311.75	7.19	9.81	36.38	1.23	0.05	−95.55
Zhejiang	63.89	45.26	−29.16	0.08	1.36	1621.93	2.22	0.35	−84.29	7.84	166.33	2020.53	40.70	10.00	−75.43	5.44	0.21	−96.23
Anhui	64.49	51.08	−20.80	0.06	0.34	446.85	0.72	0.22	−68.96	4.63	43.55	840.10	20.11	25.54	27.00	2.92	0.16	−94.52
Fujian	58.93	45.24	−23.24	0.01	0.37	6669.91	0.06	0.15	129.83	7.93	51.61	550.88	23.67	7.76	−67.21	4.70	0.93	−80.19
Jiangxi	85.08	70.83	−16.75	0.02	0.56	3447.95	0.31	0.06	−79.99	6.09	37.97	523.13	20.58	14.40	−30.05	1.69	0.05	−96.92
Shandong	42.48	28.12	−33.82	0.25	5.77	2223.91	1.88	0.10	−94.68	3.48	14.97	329.73	6.72	6.92	2.94	0.55	0.03	−93.82
Henan	73.11	55.16	−24.56	0.08	0.32	281.91	2.37	0.64	−72.83	4.30	36.70	752.95	16.17	20.95	29.61	1.52	0.02	−98.62
Hubei	84.75	63.49	−25.09	0.03	0.46	1553.63	0.93	0.60	−35.57	3.71	92.56	2392.72	39.65	26.31	−33.65	2.69	0.07	−97.48
Hunan	88.59	78.75	−11.11	0.04	2.90	7032.87	0.34	0.16	−53.31	7.10	88.19	1141.66	30.31	44.36	46.36	3.35	0.29	−91.42
Guangdong	99.51	64.81	−34.87	0.02	2.22	12,488.53	0.67	0.29	−56.25	7.37	165.08	2139.33	30.60	20.68	−32.43	1.20	0.71	−40.59
Guangxi	97.01	82.82	−14.63	0.01	0.36	4273.88	0.05	0.05	−4.42	3.63	44.03	1114.21	41.24	32.73	−20.62	2.08	2.28	9.95
Hainan	109.66	85.05	−22.43	0.00	0.27	-	0.03	0.10	187.40	4.07	31.82	681.12	72.81	80.06	9.96	0.32	1.38	324.94
Chongqing	89.90	73.37	−18.39	0.05	7.05	13,343.58	0.30	0.18	−39.46	3.54	18.89	433.23	50.98	21.51	−57.80	2.13	0.18	−91.77
Sichuan	81.74	57.34	−29.85	0.24	1.56	537.39	0.71	0.17	−76.91	1.98	10.28	419.82	16.76	18.21	8.68	2.57	0.23	−90.99
Guizhou	129.14	114.06	−11.68	0.06	1.51	2390.79	0.22	0.03	−85.94	3.45	9.70	181.39	22.48	27.26	21.26	1.42	0.33	−76.93
Yunnan	55.60	59.61	7.21	0.07	0.19	162.57	0.22	1.40	537.97	2.45	14.51	492.15	30.30	22.90	−24.42	2.07	0.49	−76.30
Tibet	118.34	166.66	40.84	0.00	0.03	-	1.31	0.53	−59.25	2.24	0.86	−61.62	15.27	31.68	107.38	60.00	0.24	−99.60
Shaanxi	67.91	55.90	−17.68	0.15	6.20	4076.18	2.20	0.19	−91.22	2.10	37.64	1690.19	21.77	21.83	0.30	3.40	0.16	−95.32
Gansu	90.64	38.71	−57.30	0.30	1.11	269.72	2.53	0.21	−91.74	8.83	30.94	250.46	20.49	20.92	2.14	5.27	0.09	−98.34
Qinghai	87.35	140.33	60.65	0.05	0.08	55.23	5.42	0.50	−90.75	1.99	18.43	825.48	23.83	34.93	46.58	12.79	0.22	−98.30
Ningxia	57.39	36.73	−36.00	0.21	0.25	19.92	1.38	0.34	−75.48	22.90	18.95	−17.26	86.84	17.94	−79.34	10.51	0.04	−99.58
Xinjiang	164.46	304.94	85.42	0.77	1.65	113.08	1.43	0.36	−74.48	4.73	25.54	440.53	23.46	19.45	−17.09	3.95	0.12	−96.99
Whole mainland China	74.27	59.27	−20.20	0.13	1.59	1123.08	2.86	0.28	−90.21	5.37	55.09	925.88	22.40	18.65	16.74	3.23	0.28	91.33

## Data Availability

The datasets used for the study are publicly available in the China Public Health Science Statistics Center (http://www.phsciencedata.cn, accessed on 10 March 2022).
